# Identifying influential observations in Bayesian models by using Markov chain Monte Carlo

**DOI:** 10.1002/sim.4356

**Published:** 2011-09-08

**Authors:** Dan Jackson, Ian R White, James Carpenter

**Affiliations:** a MRC Biostatistics UnitCambridge, UK; b London School of Hygiene and Tropical MedicineUK

**Keywords:** Bayesian methods, generalised linear models, influence, Markov chain Monte Carlo

## Abstract

In statistical modelling, it is often important to know how much parameter estimates are influenced by particular observations. An attractive approach is to re-estimate the parameters with each observation deleted in turn, but this is computationally demanding when fitting models by using Markov chain Monte Carlo (MCMC), as obtaining complete sample estimates is often in itself a very time-consuming task. Here we propose two efficient ways to approximate the case-deleted estimates by using output from MCMC estimation. Our first proposal, which directly approximates the usual influence statistics in maximum likelihood analyses of generalised linear models (GLMs), is easy to implement and avoids any further evaluation of the likelihood. Hence, unlike the existing alternatives, it does not become more computationally intensive as the model complexity increases. Our second proposal, which utilises model perturbations, also has this advantage and does not require the form of the GLM to be specified. We show how our two proposed methods are related and evaluate them against the existing method of importance sampling and case deletion in a logistic regression analysis with missing covariates. We also provide practical advice for those implementing our procedures, so that they may be used in many situations where MCMC is used to fit statistical models. Copyright © 2011 John Wiley & Sons, Ltd.

## 1. Introduction

Measures of leverage, influence and residuals are well-established tools in data analysis. Influence refers to how sensitive inferences are to the presence of particular observations and has proved its usefulness in maximum likelihood and least squares analyses.

It is therefore of interest to explore efficient algorithms for measuring influence in Bayesian analyses. Our interest in this was motivated by examples such as the sudden unexpected death in infancy (SUDI) analysis described in detail in Section 2. Here we wish to fit a standard model and examine the usual diagnostics such as influence and residuals, but some observations have missing covariates. A natural way to incorporate these observations into the analysis is to posit a model for the missing data and use maximum likelihood, but this is computationally intensive in all but the simplest of situations. In particular, this type of approach becomes increasingly difficult as the dimension of the integrals with respect to the missing data and the parameter space over which the maximisation is performed become large.

In order to overcome these difficulties, Markov chain Monte Carlo (MCMC) 1 can be used. MCMC is a powerful but computationally intensive method for fitting Bayesian models. It has the nice feature of yielding a random sample from the posterior distribution of the parameters of interest, from which means and credible intervals can be readily calculated. Although relatively simple models present few difficulties, the iteration times for complex and hence more realistic models may be long. If the mixing is poor, many iterations can be required to obtain estimates to within an appropriate degree of Monte Carlo error. Numerical estimation of influence by directly removing observations is therefore extremely tedious at best.

Here the simplest definition of influence will be taken as the difference between whole sample parameter estimates and case-deleted estimates, where each case-deleted estimate can be obtained by removing the observation in question and refitting the model 2. Although alternative measures of influence are possible, such as distances between posterior distributions of parameters of interest and the corresponding case-deleted distributions 3, these are not developed further here.

An established way to obtain influence statistics by using MCMC is importance sampling 3, 4. Here we take advantage of the relationship between the full data posterior distribution and case-deleted posterior distributions; their ratio is the likelihood of the removed observation. Hence, case-deleted posterior summaries can be obtained from weighted summaries of the sampled values by using the importance weight which is proportional to the reciprocal of the likelihood of the deleted observation evaluated at the simulated parameter values. Although this procedure is effective in many situations, it has its limitations. If the weights are very variable, then extremely large numbers of iterations may be needed; highly unusual and hence influential observations can provide surprisingly volatile weights. Hence, the problem is that, if deleting an observation causes a big change in an estimate, then the importance sampling influence statistic depends heavily on very few MCMC draws and sometimes only one. Perhaps the other main difficulty is that importance sampling requires the repeated computation of the observations' contributions to the likelihood. This becomes particularly computationally demanding as the model becomes more complex, and as a consequence importance sampling is unfeasible for very complicated models. These concerns provided additional motivation for our work which provides influence statistics that do not make any further likelihood-based computations and, in contrast to the alternatives, do not become more computationally intensive as the model complexity increases.

The routine evaluation of influence statistics was firmly established in the context of linear regression in Cook's seminal paper 5. As emphasised by Cook, ‘On the surface it might seem that any desirability this measure [a standardised measure of influence] has would be overshadowed by the computations necessary for the determination of *n*+ 1 regressions’. However, Cook shows how influence statistics can be obtained using only quantities calculated from the original regression involving the complete sample, and this also provides the foundation of approximate influence measures for the generalised linear model (GLM) introduced by Pregibon 6 and Williams 7. Hence, approximate case-deleted estimates can be obtained for any GLM without the need to fit additional regressions. In particular, the case-deleted influence statistics for GLMs given by Williams provide the justification for the procedures for obtaining these using MCMC developed here.

Although the use of MCMC to fit models with missing data provides our motivation, our methods may be used much more generally. We do however require that the likelihood dominates the prior, so that Bayesian and likelihood-based analyses provide inferences that are in good numerical, but not necessarily philosophical, agreement. This is to ensure that the established likelihood-based influence statistics are similar to their Bayesian counterparts. Here we use models that either are a standard single-level GLM or comprise a collection of connected GLMs, so we can ignore the prior specification asymptotically. Many Bayesian models are specified in this way, and this type of approach is emphasised by the models typically fitted using WinBUGS 8 (http://www.mrc-bsu.cam.ac.uk/bugs/winbugs/contents.shtml). ‘Vague’ or ‘uninformative’ priors help to ensure that their role is negligible but, provided the sample size is sufficiently large and truly dogmatic priors are avoided, are not required by the methods and models that follow. We return to the additional issues presented by more complicated models, such as hierarchical and mixed effects models, in the discussion.

The paper is set out as follows. In Section 2, we analyse the SUDI data. In Section 3, we review existing classical/frequentist methods for obtaining influence statistics for GLMs that lead us directly to our first proposal for obtaining these. In Section 4, we develop a procedure for obtaining influences using perturbed regression parameters, which, unlike our first proposal, does not require the form of the GLM to be specified. In Section 5, we describe the results from a simulation study, and in Section 6, we explain why our methods apply to models that comprise a collection of connected GLMs. In Section 7, we apply our methods to our example and compare the results with importance sampling and direct case deletion. We conclude in Section 8 with a discussion.

## 2. Sudden unexpected death in infancy

We will examine an illustrative analysis of a case control study exploring risk factors for SUDI in families with a previous SUDI 9. We have information for 137 Care of Next Infants (CONI) infants, of whom 33 are cases. This was analysed using logistic regression with key matching variables as covariates. WinBUGS 8, via the R package BRugs, was used to perform all MCMCs.

We found four variables to be especially good predictors of death in this sample by using a logistic model: Income Deprivation Affecting Children Index (DEP; a low score indicates high deprivation), mother's age in years (MAGE), birthweight in grams (BWT) and the number of previous terminations and miscarriages (TERMIN). All covariates were centred, and continuous variables were standardised, so we fit the model


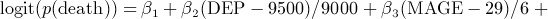
(1)


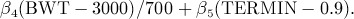


From a standard complete case maximum likelihood analysis, we obtain the following (standard errors in parentheses): 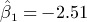 (0.55), 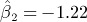 (0.47), 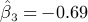 (0.29), 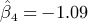 (0.33) and 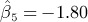 (0.58).

This complete case analysis discards 15% of the data: 21 observations have missing TERMIN values, four of which also have missing MAGE; DEP and BWT are complete. To include the incomplete cases, we fit model (1) jointly with models for the incomplete covariates. Specifically, we use a linear model for MAGE conditional on DEP and BWT and a log linear Poisson model for TERMIN on DEP, BWT and MAGE. We also use WinBUGS' truncation command to truncate TERMIN at 20. Without this, a few of the simulated TERMIN values can be unrealistically large, which has unfortunate implications for the model fit.

We place uniform priors on all regression parameters and a Gamma (0.001,0.001) prior on the precision of the variance in the linear model for MAGE. We used a burn in of 10^3^ iterations and a further 10^5^ iterations to make inferences. This MCMC took around 10 min on a Windows Terminals Server and provided 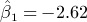 (0.54), 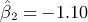 (0.42), 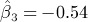 (0.28), 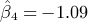 (0.33) and 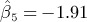 (0.60), where the estimates are given by the simulated sample means. This analysis adds further weight to the conclusion that the four variables considered are good predictors of death. We also obtained the correlation matrix of these estimates and therefore 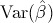. We use the notation 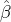 for both Bayesian and maximum likelihood estimates because we assume that they are in good numerical agreement. In particular, we use the hat notation to denote the posterior mean in all Bayesian analyses that follow, where we obtain these quantities as the sample mean of the simulated values from the MCMC.

This analysis has successfully incorporated all data but makes it difficult to assess issues such as influence. With the additional assumptions made about the missing data, coupled with the fact that there is a large amount of variation in the observed covariates, there is the natural concern that a few unusual observations might be driving the inferences. We now turn our attention to methods for deriving influences using MCMC output and begin by considering the simpler case where there is just a single GLM and no missing data.

## 3. Case-deleted estimates for generalised linear models

We condition inference on *X*, an *n* by *p* matrix of explanatory variables. The observations, denoted by *y*, are an *n* by 1 vector of conditionally independent responses. We assume for now that the data are complete but return to the possibility of missing data in Sections 6 and 7. Denote *μ*_*i*_ = *E*[*Y*_*i*_ | *X*_*i*_], where *μ*_*i*_ depends on the *i*th row of *X*, 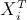, through the linear predictor 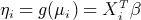, where *g*(⋅) denotes the link function and *β* is the *p* by 1 vector of regression parameters. We assume that *Y*_*i*_ is a member of the exponential family with variance *v*_*i*_ and dispersion parameter *ϕ*. Fitting the model in a Bayesian framework, and assuming that the likelihood dominates the prior, implies that the posterior means, medians and modes will all be close to the maximum likelihood point estimates, obtained using iterated weighted least squares 10, 11, with agreement as the sample size tends to infinity.

Williams 7 provides an approximation to the *i*th case-deleted maximum likelihood estimate, 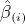, obtained by using the complete sample estimate 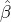 as an initial value and one step of the weighted least squares algorithm:


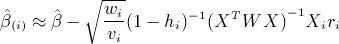
(2)

where 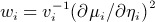, *W* = diag(*w*_*i*_), *r*_*i*_ denotes the residual (*y*_*i*_ −*μ*_*i*_) and *h*_*i*_ is the *i*th diagonal element of the ‘hat’ matrix *H* = *W*^1/2^*X*(*X*^*T*^*WX*)^−1^*X*^*T*^*W*^1/2^; all terms on the right-hand side are evaluated at the complete sample estimates. Let *θ*_*i*_ denote the canonical parameter for the regression. The density of *y*_*i*_ is exp((*b*(*θ*_*i*_) + *y*_*i*_*θ*_*i*_)/*ϕ*+ *c*(*y*_*i*_,*ϕ*)), where *μ*_*i*_ = −*b*^′^ (*θ*_*i*_) and *v*_*i*_ = −*ϕb*^″^(*θ*_*i*_). Furthermore, the matrix 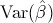 is approximately (*X*^*T*^*WX*)^−1^.

We suggest omitting the term involving *h*_*i*_ in (2). The sum of the positive *h*_*i*_ equals the number of regression parameters *p*, and we assume that the sample size *n* is much greater than *p*. Hence, although the relative magnitudes of *h*_*i*_ play an important role in determining the observations' leverages, they are much less important when obtaining influence statistics using (2); (1 −*h*_*i*_) ≍ 1 for all *i* in large samples with no grossly outlying values. With this simplification and substitution of *∂μ*_*i*_/*∂η*_*i*_ = *∂θ*_*i*_/*∂η*_*i*_ ×*∂μ*_*i*_/*∂θ*_*i*_, where *∂μ*_*i*_/*∂θ*_*i*_ = *v*_*i*_/*ϕ*, Williams' formula (2) becomes


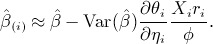
(3)

This further approximation is useful as we routinely obtain 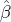 and 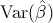 when fitting the model to the full dataset. In particular, for the SUDI data, we have already obtained 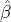 and 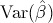 as described and reported in Section 2.

Any procedure for obtaining the rest of the right-hand side of (3) may be used to provide approximate case-deleted estimates. In particular, we can obtain influence statistics, 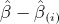, directly from (3). If the canonical link is used, *∂θ*_*i*_/*∂η*_*i*_ and (3) simplifies further.

### 3.1. Our first proposal

Assuming a particular GLM, and that the impact of the prior specification is negligible, we propose additionally defining and storing the simulated values of


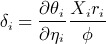


when running the MCMC. The derivatives *∂θ*_*i*_/*∂η*_*i*_ can be evaluated in terms of *η*_*i*_ and are unity if a canonical link has been adopted. Hence, the *δ*_*i*_ are easily computed in terms of the covariates, β and ϕ. Replacing *δ*_*i*_ with its estimate (the mean of the simulated values) from the MCMC in (3), and 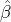 and 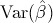 with theirs, immediately yields an approximate influence for the *i*th observation. All of the unobserved quantities that we define in this article are evaluated at every iterate of the MCMC, including derivatives, and so all of these vary between MCMC draws, but only the estimates 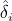, 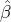 and 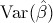 are used to compute influence statistics in (3) when using our first proposal. An assessment of the MC error for 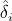 is equally important, but just as straightforward, as for 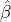 when obtaining influence statistics in this way.

## 4. Obtaining influence statistics using perturbations

Our first proposal avoids any further evaluation of the likelihood and so can be expected to be more computationally efficient than importance sampling but requires the form of the GLM to be known. In this section, we propose a second method based on perturbing the regression parameters. Although we use the theory of GLMs to justify the procedure, ultimately we use just the variance/covariance matrix of the complete sample point estimates and the posterior means of the perturbations.

We will define an *n* by *p* random matrix *ε* of ‘perturbations’ and replace the *j*th regression coefficient in the model, for the *i*th observation, by *β*_*ij*_ = *β*_*j*_ + *ε*_*ij*_. The entries *ε*_*ij*_ are to be given independent (of each other, *β* and *ϕ*) normal priors with mean 0. Otherwise, the regression is to be fitted using MCMC in the usual way. This essentially introduces a random effect component (over observations) on the regression parameters. By making the prior precisions of the random perturbations extremely large, we obtain approximately the same estimates 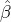 and 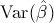 as in the usual perturbation-free regression model. We use the quantities *σ*_*ij*_ to denote the prior standard deviations of *ε*_*ij*_.

We next show in the case of a GLM that the posterior distributions of the *ε*_*ij*_ relate to influential outcomes in the model. The intuition is that, if an observation is directly influential for a particular parameter, then the corresponding posterior *ε*_*ij*_ distribution will move further from zero than its less influential counterparts, reflecting the ‘pull’ or influence that this observation exerts on the fitted model.

### 4.1. The mathematical consequences of introducing the perturbations

Let 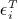 denote the *i*th row of the perturbation matrix. With the assumption that each of the *n* observations, conditional on all *ε*_*ij*_, *β* and *ϕ*, are independent (equivalent to assuming that they are exchangeable in the original model),


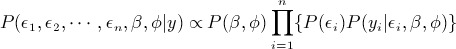
(4)

Note that we include the prior *P*(*β*,*ϕ*) for completeness, but we assume that its role is negligible in practice. We show in the Appendix that, provided the prior perturbation variances (denoted by 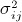) are made sufficiently small, the posterior mean of *ε*_*ij*_ is given by 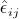, where


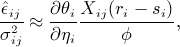
(5)


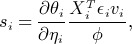


*X*_*ij*_ denotes the *j*th entry of 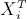, and all quantities are on the right-hand side of (5) are evaluated at their posterior modes.

### 4.2. An approximation

We assume that the magnitude of the posterior mode of *s*_*i*_ is small in relation to that of *r*_*i*_. We can check this, provided that the form of the GLM is known, as shown for our example in Section 7. As 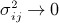, *s*_*i*_ → 0, and we can ignore *s*_*i*_. Hence,


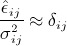
(6)

where *δ*_*ij*_ is the *j*th entry in *δ*_*i*_ and is evaluated at full sample estimates in exactly the same way as in Williams' approximation for case-deleted estimates. For models where the variance *v*_*i*_ is unbounded, extra care must be taken to ensure that *s*_*i*_ is small in relation to the residuals because *s*_*i*_ is linear in *v*_*i*_. For example, for datasets with large Poisson counts, very small 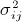 may be required to ensure that we can ignore *s*_*i*_ in this way.

We can therefore obtain the term *δ*_*i*_ ≈ {*∂θ*_*i*_/*∂η*_*i*_}(*X*_*i*_*r*_*i*_/*ϕ*) using MCMC by adding the perturbations and monitoring the simulated *ε*_*i*_ or equivalently by defining the random variables 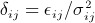 and monitoring the *δ*_*i*_. With the combination of (6) and (3), the case-deleted estimates are


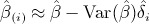


from which we can obtain the influences, 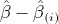.

### 4.3. Implementation and Monte Carlo error

A practical issue is that, although it is possible to implement this method in a single stage as presented above, this is computationally demanding and frequently results in ‘trap errors’ in WinBUGS. We suggest a two-stage procedure that avoids these difficulties in practice. In the first stage, the original model is fitted without perturbations in the usual way in order to obtain 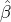, 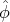 and 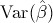.

In the second stage, to simplify the MCMC algorithms, we suggest constraining *β* and *ϕ* to their estimates from the first stage and then introducing the perturbations. With *β* and *ϕ* fixed in this way, the posterior for the *ε* vectors becomes (4) with *P*(*β*,*ϕ*) = 1 and *P*(*y*_*i*_ | *ε*_*i*_,*β*,*ϕ*) replaced by 
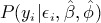
, and a similar 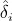 is obtained. This simplification is made purely for computational convenience because suitable 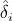 are evaluated regardless of whether a one-stage or a two-stage procedure is adopted.

Now that *β* and *ϕ* are to be held fixed in this second stage, the only observation which is used to update the prior of *ε*_*i*_, and therefore *δ*_*i*_, is *y*_*i*_. Hence, we can add the *ε*_*i*_ to a single observation (but to all regression coefficients) at a time, providing *y*_*i*_ as the only observation, and run *n* additional analyses. In practice, we found it convenient and computationally efficient to add the perturbations in this manner, so that 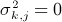 for all *k* ≠ *i* when obtaining the *i*th influence statistic. It is then a very simple task to update the vector *ε*_*i*_ *en bloc* using a Gibbs' sampler, and, because there are no other random variables, we simulate directly from the posterior *ε*_*i*_ | *y*_*i*_ and the simulated *ε*_*i*_, and hence *δ*_*i*_ are independent. The resulting MCMC mixes and converges extremely rapidly.

Monte Carlo error is inherent in the influences obtained using equation (6), which is a little more difficult to assess because of the indirect manner in which influence statistics are obtained when using the perturbations. The error in 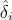 is approximately normally distributed, centred at the origin with 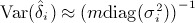, where 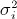 is the vector of 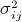 associated with observation *i*, and *m* is the number of iterations. This is because *δ*_*i*_ has a prior variance of 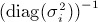, and in the two-stage approach, the simulated *δ*_*i*_ are independent as explained above; the posterior variance of *δ*_*i*_ is therefore very similar to its prior. Hence, the Monte Carlo error of the influence of the *i*th observation, obtained as 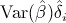, is approximately 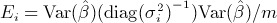, so that a good indication of this Monte Carlo error can easily be obtained. Small values of 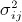, which improve the accuracy of the approximations, also increase Monte Carlo error of the influence statistics, and some kind of tradeoff is needed in practice. If the perturbations are made quite large in this Monte Carlo error/approximation tradeoff, then they do not affect the primary analysis because this is performed in the first ‘perturbation free’ stage. Large entries of 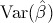 also have an unfortunate implication for the Monte Carlo error, so we suggest centring data before fitting models to reduce the variance of intercept terms.

Although an advantage of this approach is that we merely require the model to be some (unspecified) GLM, the form of the GLM does have implications for how small the perturbations' variances have to be, which in turn has implications for *m*. In practice, it may be desirable to use a variety of small *σ*_*ij*_ and large *m* in order to determine if reliable influence statistics have been obtained. Alternatively, and if known, the properties of the GLM in question can be examined and suitable criterion chosen. Despite this, the difficulties associated with choosing appropriate values of *σ*_*ij*_ and *m*, so that both the approximations are accurate and the MC error is small, are a disadvantage of this method. Our first proposal may therefore be considered preferable in situations where the form of the GLM is known.

## 5. A simulation study

In order to assess the accuracy of the two proposed methods, we performed a simulation study. Here we simulated 100 datasets, each with *n* = 100 observations, by using the linear model *Y* ∼ *N*(*β*_0_ + *β*_1_*x*_1_ + *β*_2_*x*_2_,1), where *β*_0_ = 0, *β*_1_ = 1 and *β*_3_ = 3. Burn ins of 10^3^ iterations were used. A further 10^5^ iterations were used when implementing our first proposal, and *m* = 10^6^ iterations were used when obtaining influences using the perturbations to ensure that these had stabilised.

By using normally distributed data, exact frequentist influence statistics 5 can be obtained which provide a ‘gold standard’ to compare our methods with. Because we used a linear model with a canonical link, our simulation study does not assess the accuracy of the approximations described in the Appendix; rather, it assesses how well our proposals work when these approximations are valid. Flat priors over very wide ranges were used for the *β* parameters, and a Gamma (0.001,0.001) prior was used for the precision of the variance.

Perturbations with *σ*_*ij*_ = min(0.05/ | *X*_*ij*_ |, 1) were used. These values have been found to ensure as large perturbations as possible are used, without compromising the accuracy of any approximation, when a canonical link is used in either a linear (with variance of around 1 or greater) or logistic regression involving a moderate number of parameters. For a GLM with a canonical link, the approximations described in the Appendix only require that values of 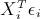 are small and the posterior modes of the *s*_*i*_ are small in relation to the residuals *r*_*i*_; 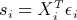 for a standard linear regression. For example, for *p* = 6 as in our main example, using | *X*_*ij*_ | *σ*_*ij*_ = 0.05 ensures that 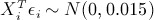, so that probable values of 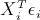 are small on the log odds scale. Numerical difficulties (‘trap errors’ in WinBUGS) can, however, be obtained if instead *σ*_*ij*_ = 0.05/ | *X*_*ij*_ | is used because this results in very large *σ*_*ij*_ if covariates are close to zero and hence potential numerical instability. We suggest considering the use of *σ*_*ij*_ = min(*a*/ | *X*_*ij*_ |, *b*) in practice and have found *a* = 0.05 and *b* = 1 to be suitable for our examples.

By combining the influence statistics across all simulated observations and datasets, we have 100 ×100 = 10 000 exact frequentist influence statistics for each regression parameter to compare our methods with. Both our proposed methods provided influence statistics that were in good agreement with these standard influence statistics. To give an indication of this, the coefficients of the least squares regression lines of the proposed influence statistics on the standard frequentist influence statistics are given for each parameter in Table 1, where the correlations between the proposed and standard influence statistics are also shown. The correlations are given to five decimal places so that they are distinguishable. The gradients of the regression lines are all between 0.95 and 0.99 suggesting that, compared with the exact frequentist influences, the proposed methods have a slight tendency to understate the influence of observations in this simulation study, but the overall picture is that all three methods are in good agreement. This tendency can be explained by the omittance of the (1 −*h*_*i*_) terms in (2), so this will lessen in larger samples.

**Table:** Some results from the simulation study: *c*_1_ and *m*_1_ denote the intercepts and gradients of the least squares regression lines of influence statistics from our first proposal (Section 3.1) on the frequentist influence statistics; *ρ*_1_ denotes the correlation between these influence statistics. *c*_2_, *m*_2_ and *ρ*_2_ denote these same quantities for influences obtained using perturbations

Parameter	*c*_1_	*m*_1_	*ρ*_1_	*c*_2_	*m*_2_	*ρ*_2_
*β*_0_	0.0000	0.9832	0.99979	0.0002	0.9761	0.99978
*β*_1_	0.0000	0.9648	0.99964	0.0000	0.9578	0.99963
*β*_2_	0.0000	0.9726	0.99962	−0.0001	0.9655	0.99957

## 6. Obtaining influences for models comprising a collection of connected generalised linear models and in situations where there are missing data

The above analysis assumes a single GLM (with complete data), but as explained in the introduction, Bayesian models are commonly defined as a collection of connected GLMs. For example, the model for the SUDI data is such a collection of three GLMs, where the logistic regression of death on all the covariates is of real interest. There are also some missing data, which motivated our methods as we explained in the introduction.

Our methods are, however, immediately applicable to any GLM component within the full model. When implementing our first proposal (Section 3.1), we simply define *δ*_*i*_ = {*∂θ*_*i*_/*∂η*_*i*_}(*X*_*i*_*r*_*i*_/*ϕ*) for the GLM for which influences are desired and combine these with the resulting regression estimates, 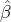 and 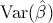, for this same GLM. Neglecting any prior dependence between regression parameters that might have been specified, and if there is no missing data, the parameter estimates for each GLM are independent, and hence, the standard theory described in Section 3 ensures that influence statistics are obtained from our methods.

There is, however, the issue of missing covariates, which is ignored in the above argument. This is in fact of little concern provided the fraction of missing data is not too severe because our procedures average the influences over the posterior distributions of any missing data, and hence, suitable influences are obtained. This way of handling missing data may be considered an advantage of our methods. We may therefore handle missing data by entering them as ‘NA’ in the usual way when using WinBUGS, for example.

The perturbations are justified as they obtain *δ*_*i*_ in a different way and hence are also appropriate. Here we constrain all parameters to their estimated values after fitting the model comprising a collection of connected GLMs in the first stage and then add the perturbations to the GLM in question and monitor the simulated *δ*_*i*_ in the same manner as before.

## 7. Influence statistics in the sudden unexpected death in infancy analysis

We used the proposed methods, and the alternatives, to estimate the influences in the SUDI analysis. Our first proposal is suitable because the form of the model of interest (the logistic regression for the probability of death) is known, but our second proposal will also be used so that the results can be compared.

There are some missing covariates, but otherwise model (1) is a standard logistic regression. We therefore also monitored 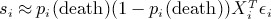 when adding the corresponding perturbation to ensure that the role of *s*_*i*_ is negligible in (5). The average absolute posterior mean of 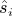 was just 0.0006, and its maximum was 0.0024. These are small values in relation to the residuals resulting from logistic regression, and we are reassured that approximation (6) is accurate. A burn in of 10^3^ iterations was used. A further 10^5^ iterations were used to obtain influences using our first proposal, and the influences from the perturbations-based method appeared to stabilise (small MC error) using *m* = 10^6^. Perturbations with | *X*_*ij*_ | *σ*_*ij*_ = 0.05 were initially used, but some numerical difficulties were encountered for the three data points where the mother's age was 29 years; this corresponds to a covariate of zero in the regression as written above and, hence, with the decision to use | *X*_*ij*_ | *σ*_*ij*_ = 0.05, an infinite *σ*_*ij*_. In fact, covariates were centred at exact means rather than the approximate ones given above, but the three very large *σ*_*ij*_ arising from this strategy presented problems. Following the procedure used in the simulation study to avoid these problems, *σ*_*ij*_ was therefore reduced to unity when used in conjunction with the mother's age covariate for these three observations.

### 7.1. Comparison of methods

**Figure 1:**
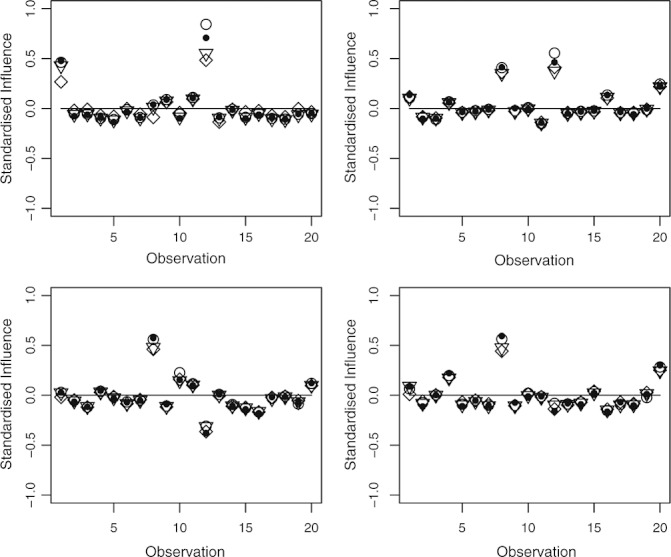
Standardised influence statistics for the first 20 observations. The top two panels show influences for *β*_2_ and *β*_3_, whilst the bottom two panels show these for *β*_4_ and *β*_5_. Solid points show the influence statistics obtained by case deletion; hollow circles show the corresponding estimates obtained by importance sampling; triangles show these using our first proposal (Section 3.1); and diamonds show influences obtained using perturbations.

A gold standard influence analysis was performed by removing each of the 137 observations in turn. Only 20 000 iterations were used to obtain estimates for each case-deleted sample to reduce the time taken (to around 5 h). The resulting case-deleted influences were standardised (divided by the standard error of the corresponding complete sample parameter estimate) and are shown as solid points in Figure 1 for the first 20 observations, where standardised influences for *β*_2_ and *β*_3_ are shown in the top two panels and those for *β*_4_ and *β*_5_ are shown in the bottom two panels. The 95% intervals describing Monte Carlo uncertainty, from normal approximations, are only slightly wider than the solid points which can thus be taken to be accurate. Hollow circles show the corresponding estimates obtained by importance sampling 3, 4; triangles show these from our first proposal (Section 3.1), and diamonds show influences obtained using perturbations (our second proposal).

All three approximations perform least well for the more influential observations but are generally effective in obtaining influences. It is to be expected that large influences are obtained with the least precision, as these provide the biggest challenge to Williams' approximation (which two of the methods are based on) and the most volatile weights for importance sampling. Very similar results were obtained for the remaining 117 observations. Note in particular that all methods successfully detected the influential observation 12 for *β*_2_. It is not surprising that observation 12 is influential; this has the highest DEP score of the cases and one of the oldest MAGE values. Cook 5 suggests, in the context of linear regression, that if removing an observation results in an estimate at the edge of 50% of the whole sample confidence region, then this ‘may be cause for concern’. Observation 12 can therefore be interpreted as being worryingly influential for *β*_2_ but not excessively so.

Because all the procedures were successful, it is perhaps the additional computational burden required to obtain influences that determines which is to be recommended in practice. Importance sampling doubled the overall simulation time (from around 10 to 20 min). Using our first proposal only required an extra 5 min. Even for this relatively simple example, our first proposal was much faster than importance sampling. This relative efficiency will increase as the model complexity, and hence the difficulties associated with evaluating the observations' contributions to the likelihood, also increases.

The perturbations-based method required around 30 s for each observation, so that around an hour of computing time was needed to obtain influence statistics. Nevertheless, this method has the advantage that computation time does not increase with model complexity in the way it does with importance sampling and may prove useful for models where the precise form of the GLM cannot be specified. Indicative WinBUGS code for performing the analysis is available from the first author on request.

## 8. Discussion

This paper has developed novel ways of approximating established influence statistics, as used in classical methods, so that they can be used in the context of Bayesian analyses using MCMC. The two new approaches involve storing additional quantities and then use standard MCMC output to recover Williams' influence statistic. The type of analysis performed provided the motivation for this work, which can be applied much more generally. We have not, however, attempted to answer the much more difficult question of whether or not removing particular observations changes the conclusions qualitatively. Furthermore, we have not asked the even more difficult question of what we should do if we discover that an observation is very influential.

Direct case deletion, although easily implemented, is not a feasible option unless models can be fitted extremely quickly; even for the relatively simple model considered here, with just over a hundred observations, this took several hours. Importance sampling is a generally viable but fairly computationally intensive method for the type of models we have considered but becomes computationally prohibitive as the model becomes more complex and the repeated computation of the likelihood becomes less feasible. Even for our relatively simple example, the computational advantages of our first proposal are apparent. If any of the procedures that avoid direct case deletion highlight extremely influential observations, and an accurate indication of their influence is desired, it is necessary to remove the offending observations and refit the model. This is because all the approximate influences are less accurate for more influential observations. All that is typically needed in practice, however, is the identification of influential observations, and an indication of the extent of this influence and our procedures can be used for these purposes.

We have assumed that the model is a collection of connected single-level GLMs. Our methods might also be considered when the part of the model of interest is either known to be such a GLM or, at the very least, thought to behave similarly to one when using the perturbations. Bayesian Hierarchical models, where unobserved random effects present further issues 12, do not fit directly into our framework, however. Despite this, Hodges' 13 proposals for the assessment of case influence for hierarchical models, in his Section 4.4, bear some similarities to ours. Extensions or variations of our methods might be especially useful in this context and may form the subject of future work.

In particular, because the perturbations have the intuition that influential observations will exert more ‘pull’, it would be especially interesting to see if these are also useful for these and other types of statistical model. A difficulty in using the perturbations in practice is that we must ensure that all the various approximations used are appropriate. For models where the variance is unbounded, extra care must be taken to ensure that *s*_*i*_ is small in relation to the residuals because this is linear in *v*_*i*_. Furthermore, if a canonical link has not been used, we have a further criterion that must be satisfied in terms of the derivatives of the canonical parameter and the linear predictor.

In conclusion, we have proposed two new methods for estimating the influence of observations when fitting models by using MCMC. We have shown how these are related and given practical guidance. Both proposals have been shown, in an example, to give accurate measures of influence. Both proposals avoid any further computation of the likelihood and so can be used in some situations where the complexity of the model renders case deletion and importance sampling unfeasible.

## Appendix

We assume a GLM so that the observations *y*_*i*_ follow distributions from the natural exponential family with canonical parameter *θ*_*i*_, so *P*(*y*_*i*_ | *θ*_*i*_) ∝ exp((*b*(*θ*_*i*_) + *yθ*_*i*_)/*ϕ*). With the perturbations and an arbitrary link function, we have , where *f*(⋅) is a strictly increasing function; if this is the identity function, then we have assumed the canonical link. We also have the standard results

(7)

and

(8)

Using independent normal priors for the *ε*_*ij*_, and noting that all *ε* prior distributions are independent, gives

(9)

The distributional assumptions of the GLM, with the perturbations introduced, gives

(10)

The product of (9) and (10) gives the posterior density of *ε*_*i*_ to within a constant of proportionality. Taking the logarithm of the resulting density and differentiating with respect to *ε*_*ij*_ gives

(11)

where *X*_*ij*_ denotes the *j*th entry of *X*_*i*_^*T*^; *f*^′^ (⋅) = *∂θ*_*i*_/*∂η*_*i*_ and *b*^′^ (⋅) are the derivatives of functions *f*(⋅) and *b*(⋅). In order to obtain the posterior distribution of *ε*_*ij*_, a series of approximations are required.

### Approximation 1

We use the linear approximation  and note that this is exact if a canonical link is used and valid as  for all *j* = 1, ···, *p*. Hence,  where the partial derivatives are evaluated at . We further assume . Again as , this criterion is satisfied, and this is exact if a canonical link is used. Assuming sufficiently small perturbations, we approximate  in (11).

### Approximation 2

We use two linear approximations, firstly for the function *f*(⋅), taken around , and then for *b*^′^ (⋅), taken around , to approximate . From (7) and (8), this is equivalent to , where the partial derivative and the moments of *Y*_*i*_ are evaluated at . The first of these linear approximations requires small , and the second requires small . For a canonical link, these requirements are the same, and the first linear approximation is exact. The second linear approximation is exact for a linear model and canonical link. All these approximations are justified for any GLM as , however.

### An approximate derivative

Upon making approximations 1 and 2, (11) becomes

(12)

where *r*_*i*_ denotes the residual (*y*_*i*_ −*μ*_*i*_), and . We obtain the posterior mode by setting derivatives (12), *j* = 1, ···, *p*, to zero. Hence, denoting (for the moment) the posterior mode of *ε*_*ij*_ using the ‘hat’ notation, we obtain (5). A linear approximation for *b*(⋅) in (3) further shows that the posterior of the *ε*_*i*_ are also approximately normal. Hence, the ‘hat’ notation in (5) can be taken to refer to any conventional measure of location, so we take this to indicate the posterior mean.
